# In Situ FTIR Spectroscopic Monitoring of the Formation of the Arene Diazonium Salts and Its Applications to the Heck–Matsuda Reaction

**DOI:** 10.3390/molecules25092199

**Published:** 2020-05-08

**Authors:** K. Sateesh Reddy, Bandi Siva, S. Divya Reddy, N. Reddy Naresh, T. V. Pratap, B. Venkateswara Rao, Yi-An Hong, B. Vijaya Kumar, A. Krishnam Raju, P. Muralidhar Reddy, Anren Hu

**Affiliations:** 1 Technology Development Center, Custom Pharmaceutical Services, Dr. Reddy’s Laboratories Ltd., Hyderabad 500049, India, prataptv@gmail.com (T.V.P.); 2 AU College of Engineering (A), Andhra University, Visakhapatnam 530003, India; profbattula@gmail.com; 3 Laboratory for Bioanalytical Chemistry, Institute of Chemistry, University of Neuchatel, Avenue de Bellevaux 51, CH-2000 Neuchatel, Switzerland; bandishiva2008@gmail.com; 4 Department of Pharmacy, University College of Technology, Osmania University, Hyderabad-500007, TS, India; divya4474@gmail.com; 5 Department of Chemistry, University College of Science, Osmania University, Hyderabad-500007, TS, India; nareshnadipolla@gmail.com; 6 Department of Laboratory Medicine and Biotechnology, College of Medicine, Tzu-Chi University, Hualien 97004, Taiwan; amyhung840809@gmail.com; 7 Department of Chemistry, Nizam College, Osmania University, Hyderabad 500001, TS, India; vijaychemou@gmail.com (B.V.K.); krishnamrajua@gmail.com (A.K.R.)

**Keywords:** in situ FTIR-ATR, arene diazonium salt, Heck–Matsuda reaction

## Abstract

This study depicts the use of a fiber-optic coupled Fourier transform infrared spectroscopy-attenuated total reflection (FTIR-ATR) probe for the in-depth study of arene diazonium salt formation and their utilization in the Heck–Matsuda reaction. The combination of these chemical reactions and in situ IR spectroscopy enabled us to recognize the optimum parameters for arene diazonium salt formation and to track the concentrations of reactants, products and intermediates under actual reaction conditions without time consuming HPLC analysis and the necessity of collecting the sample amid the reaction. Overall advantages of the proposed methodology include precise reaction times as well as identification of keto enol tautomerization in allylic alcohols supporting the ‘path a’ elimination mechanism in the Heck–Matsuda reaction.

## 1. Introduction

**Scheme 1:**
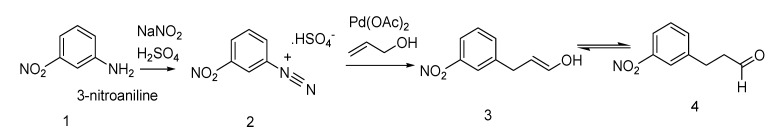
Overall reaction of the formation of diazonium salts and the Heck–Matsuda reaction.

Owing to their strong reactivity and various transformations, arene diazonium salts are common, readily synthesized and incredibly important intermediates in organic synthesis [1]. Multiple well-known named reactions allied to arene diazonium salts have been developed since their discovery (Scheme 1) [2]. To date, arene diazonium salts have been used in Pd-catalyzed cross coupling reactions for the development of C-C bonds as reactive aryl halide surrogates [3,4,5]. The greater electrophilicity of diazonium salts is due to the presence of a N_2_ group, thereby favoring mild reaction conditions. Due to their exothermic nature, they are synthesized in cold aqueous solutions [6]. Exothermic reactions, especially during scale up, present safety hazards like high pressure, fire and explosion, besides reduction of product purity and yield. Insufficient monitoring of these reactions leads to safety concerns. Over the past few decades, online analytical technologies related to process analytical technology (PAT) and quality by design (QbD) concepts have drawn the attention of researchers to control chemical processes [7,8]. Several reports reveal that the utilization of PAT technologies can provide vital process information in less time in the face of the challenges related with processes that are more complex. Out of these technologies, ReactIR is a significant model [9] wherein a robust attenuated total reflection (ATR) probe is inserted directly into the vessel and spectra are collected and transformed into a molecular video of the reaction [10,11,12].

In situ process FTIR together with ReactIR 45m enable a comprehensive understanding of the reaction, the recognition of process issues and the monitoring of the initiation, progress and endpoint of the reaction. The main benefits of this technique are the exclusion of time-consuming analysis and reduction of the vulnerability of workers to hazardous chemicals. Along a similar line, many approaches have been utilized in order to avoid exposure to hazardous substances. One such approach is the in-situ production of aryl diazonium salts, followed by their reaction without isolation [13].

In this article, we tried to understand and optimize the chemical reaction involving the formation of arene diazonium salts and their conversion into alkenes through a fusion of the Heck–Matsuda reaction and in situ IR spectroscopy. Tracking of the Heck–Matsuda reaction by in situ IR spectroscopy at different relative times revealed that the allylic alcohols undergo keto enol tautomerization during their conversion to aldehydes.

## 2. Results and Discussion

Diazonium salt formation is an intermediate step in many organic chemical reactions and it plays a characteristic role in the result of the applicable chemical reactions [14]. Generally, monitoring of these intermediates is not possible in real time and it may take several hours to characterize and determine its purity and yield in situations where the intermediate can be isolated [15].

**Figure 1:**
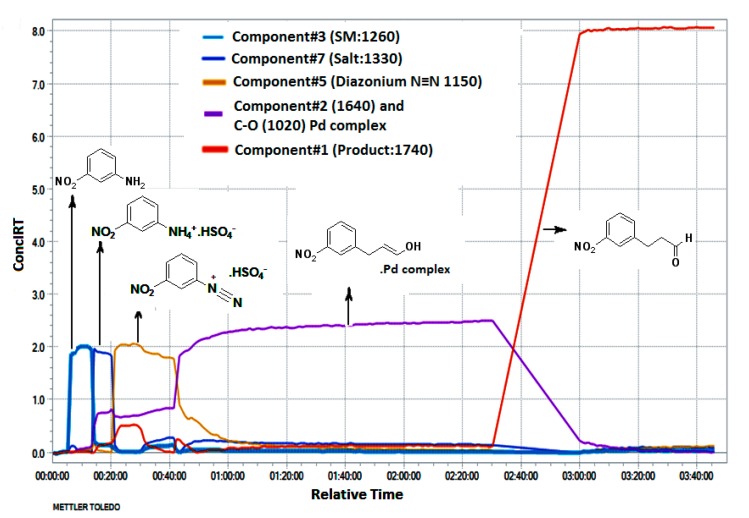
Trends of aryl diazonium salt formation followed by the Heck–Matsuda reaction and keto enol transformation, SM (starting material), ConcIRT (concentration infrared trends).

**Figure 2:**
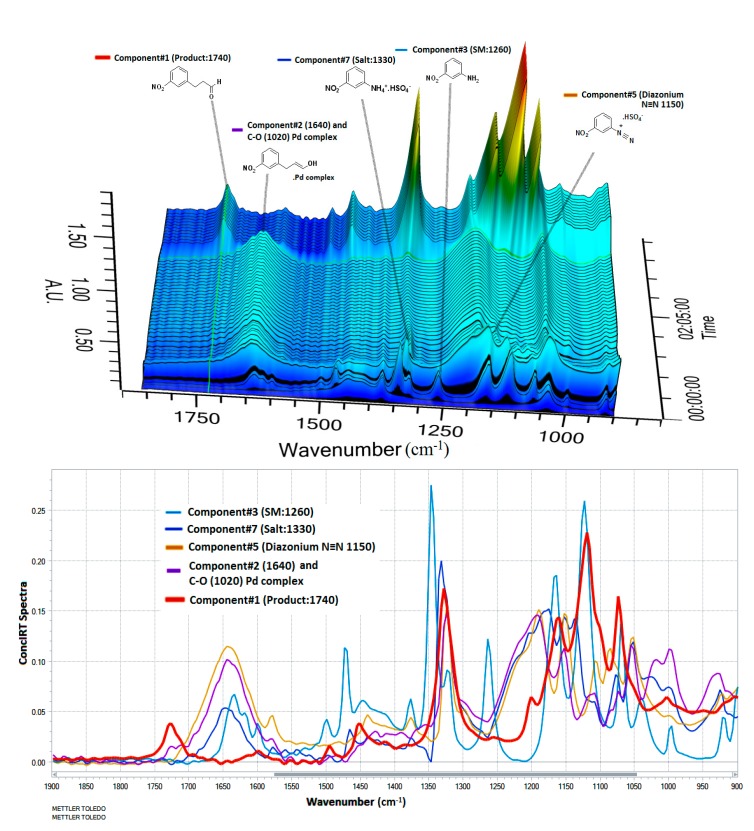
Three-dimensional surface for complete reaction and its IR spectrum. A.U.: arbitrary units.

Using FTIR as a reaction monitor can help to recognize the formation of diazonium salt intermediates along with the detection of reactant depletion and product formation. In order to ease our process control, we chose to observe the progress of the reaction using an in situ FTIR probe instrument and an automated reactor. Such coupling will allow us to better understand the issues arising throughout the reaction, like temperature variations and chemical events. Figure 1 demonstrates the in situ FTIR trend profile obtained (Scheme 1) and Figure 2 illustrates a three-dimensional (3D) surface plot of the concentration infrared trends(ConcIRT )spectra captured during the complete reaction. Aryl diazonium salts were synthesized by diazotization of the 3-nitro aniline with NaNO_2_ in the presence of an inorganic acid (H_2_SO_4_), which serves as a proton donor and a counter ion. These aryl diazonium salts were then directly used in a Heck–Matsuda reaction (Scheme 1) [13].

**Scheme 2:**
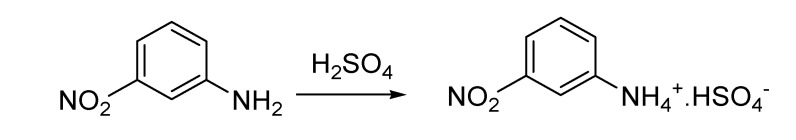
Step 1, formation of salt.

**Figure 3:**
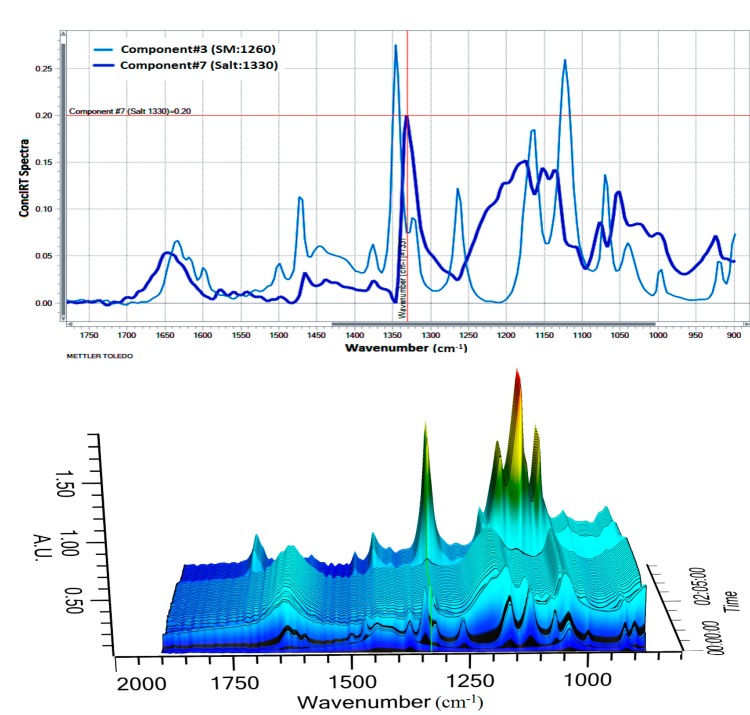
Comparison of IR spectra and three-dimensional surface of the amine (component #3 (light blue)) and its salt (component #7 (dark blue)).

**Scheme 3:**
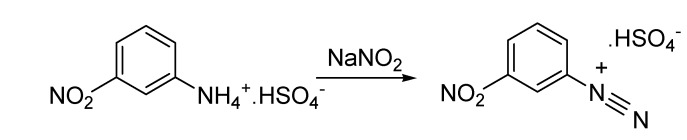
Step 2, formation of diazonium intermediate.

**Figure 4:**
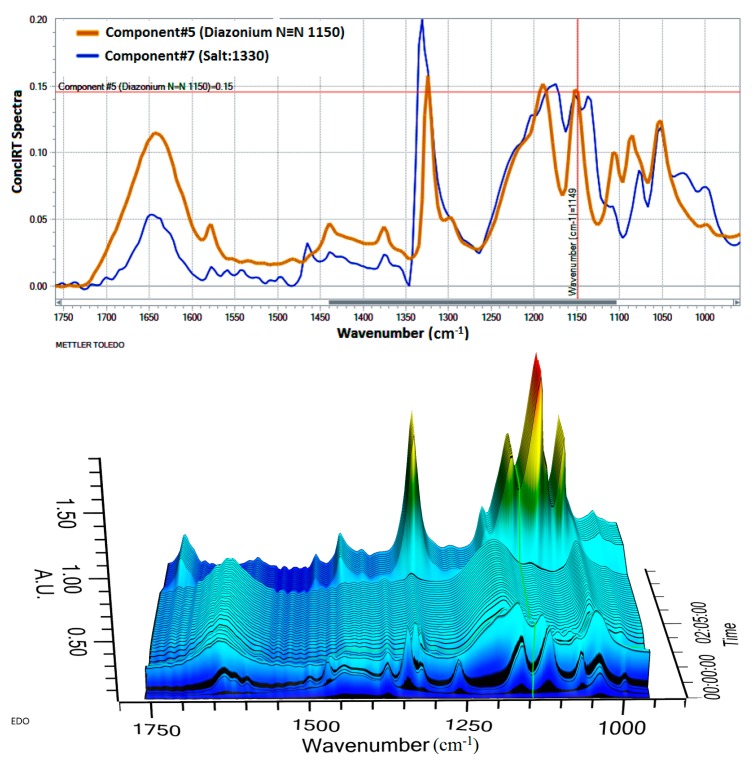
Comparison of IR spectra and three-dimensional surface monitoring of the formation of diazonium salts (component #5 (dark yellow)) from amine salt (component 7 (dark blue)).

**Scheme 4:**
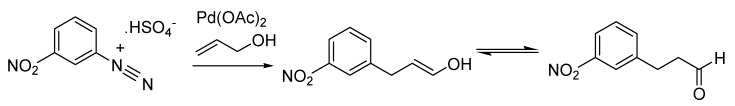
Step 3, formation of alkene product followed by keto enol tautomerism.

**Figure 5:**
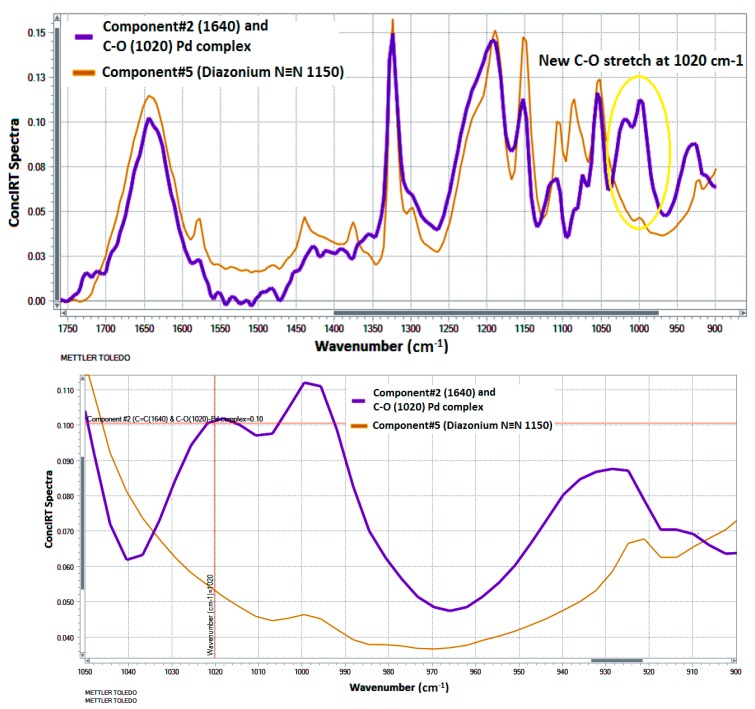
Comparison of IR spectra for the formation of the alkene–palladium complex product (component #2 (purple)) from diazonium salts (component #5 (dark yellow)).

**Figure 6:**
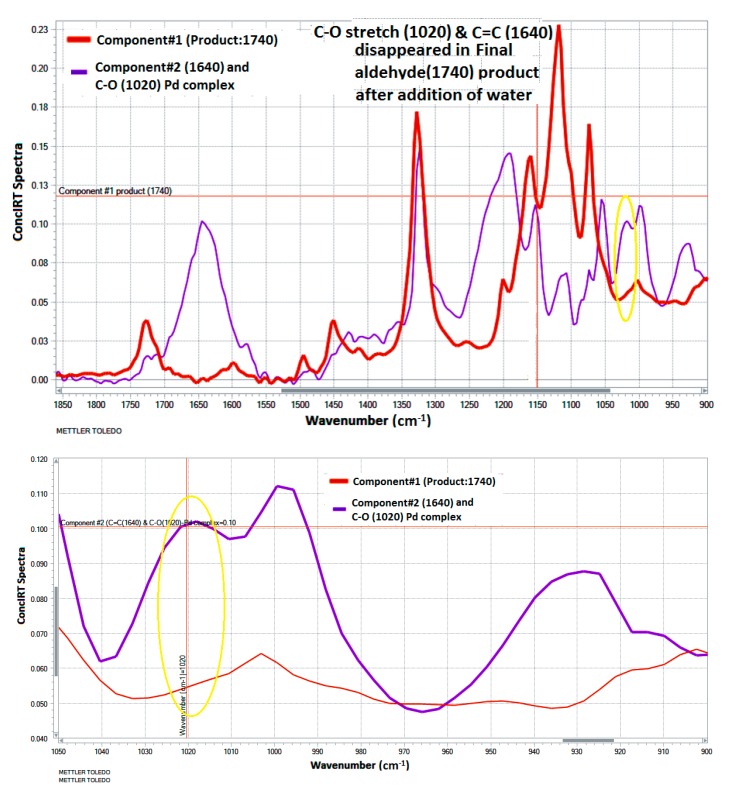
Comparison of IR spectra for the formation of aldehyde (component #1 (red)) from the alkene–palladium complex product (component #2 (purple)) (keto enol tautomerism).

**Figure 7:**
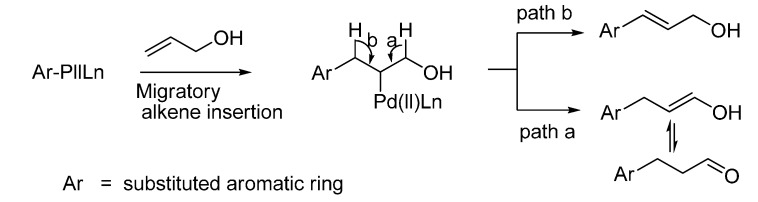
Chemo selectivity in the Heck reaction [16].

The ReactIR software permits data processing that determines the concentration of various components throughout the reaction (Figure 1 and Figure 2). As depicted in Scheme 2 and Figure 3, the addition of sulfuric acid is concomitant with the consumption of the starting material (SM), 3-nitro aniline (light blue trend line in Figure 1, component #3 signal at 1260 cm^−1^), for 5 min and the formation of the amine sulfuric acid (salt) intermediate (dark blue trend line in Figure 1, component #7 signal at 1330 cm^−1^) at 15 min. Upon the addition of sodium nitrite, this intermediate was converted to diazonium salt (Scheme 3 and Figure 4) (dark yellow trend line in Figure 1, component #5 signal at 1150 cm^−1^) at 20 min. At this stage, we added allyl alcohol along with palladium acetate; the reaction proceeded smoothly without changes until 2 h 30 min. A long time was taken to complete the Pd mechanism cycle, leading to the disappearance of the diazonium salt intermediate (Scheme 4 and Figure 5) (dark yellow trend line in Figure 1, component #5 signal at 1150 cm^−1^) and appearance of the alkene peak in the palladium complex (purple trend line in Figure 1, component #2, C-O signal at 1020 cm^−1^ and C=C signal at 1640 cm^−1^). Upon the addition of water, this alkene–palladium complex was converted to aldehyde through keto enol tautomerism. This conversion was demonstrated clearly in the IR trend (Scheme 4 and Figure 6) (red trend line in Figure 1, component #1 signal at 1740 cm^−1^), and was clearly supported by the IR spectrum, where the C=C signal at 1640 cm^−1^ and the C-O signal at 1020 cm^−1^ were disappearing and the aldehyde 1740 cm^−1^ signal was appearing (Figure 6). The reaction was finished at 2 h 30 min. Similarly, we assume that step 2 (i.e., preparation of diazonium salts) and the amount of Pd catalyst added are the most important steps for the Heck–Matsuda reaction. This aldehyde formation clearly indicates the formation of a double bond through ‘path a’ elimination, as shown in Figure 7. The product and starting material amounts were quantified at any stage of the reaction. This quantitative in situ FTIR analysis was carried out using the calibration curve method [10]. We also performed similar analysis for another example using 3-(trifluoromethyl) aniline and 2-vinylbenzoic acid as starting materials as shown in the supplementary materials (Figures S5 and S6).

Such online monitoring is, therefore, helpful for this particular step, and we can sustain the process to avoid delayed analysis and sampling during the reaction.

## 3. Materials and Methods

### 3.1. General

In this experiment, reactions were carried under a N_2_ (nitrogen) atmosphere. Reagents and solvents were purchased from commercial sources. In situ infrared spectra were acquired using a ReactIR 45m with MCT (mercury-cadmium-telluride) detector from Mettler Toledo Autochem (Columbia, SC, USA) [9]. The probe DiComp (Diamond, CO, U.S.A) was connected through a AgX 9.5 mm × 1.5 mm silver halide fiber. In all experiments, the area from 1900–900 cm^−1^ was used for analysis.

### 3.2. Instrumental Conditions and Data Processing

The instrument was fitted with Dicomp (Diamond) probes connected through a 1.5 mm AgX fiber. The probe was introduced into the reactor using custom Teflon fittings to set the probe depth in zone away from the impeller vortex. Prior to all experiments, an air background was obtained. Mettler Toledo ICIR software version 4.3 was utilized for controlling the instrument and analyzing the data. Data were acquired at an 8-wave number resolution and an automatic sample scan interval of 5 s from 1900 cm^−1^ to 900 cm^−1^.

### 3.3. Formation of Diazonium Salts and Alkene

The parent aniline (1.0 m mol) was dissolved in acetonitrile (10.0 v) into a vessel for 0–5 min and aqueous sulfuric acid (2 mmol) was slowly added from 5–15 min at 5 °C. To that solution, sodium nitrite (2 m mol) was added at same temperature at 16 min and the mixture was heated to room temperature. Palladium acetate (0.004 m mol) and allyl alcohol (1 m mol) were added at 20 min and the reaction mixture was monitored for 2 h 10 min. Finally, water was added at 2 h 30 min and compound was extracted into toluene.

## 4. Conclusions

In conclusion, the use of an in situ IR PAT tool enabled the monitoring of the formation and utilization of diazonium salts and reaction intermediates, which is vital for the successful development of this process. The completion of the arene diazonium formation reaction and its application to the Heck–Matsuda reaction was confirmed by the IR online analysis, rendering the need for HPLC/TLC analysis unnecessary. Further, the online monitoring allowed for the identification of keto enol tautomerization in allylic alcohols. The application of in situ FTIR coupled with an automated reactor was therefore critical for accurate progress control of the reaction, enabling this process to scale-up safely and reliably.

## Supplementary Materials

The following are available online at https://www.mdpi.com/1420-3049/25/9/2199/s1, Figure S1: Comparison of IR spectra with component #5 vs 26:15 min spectra vs 37:15 min spectra to understand the trend changes in figure 1, Figure S2: Comparison of IR spectra of standard vs isolated product, Figure S3: Complete IR spectra with all 7 components, Figure S4: Comparison of IR spectra of reference acetonitrile vs component 6, Figure S5: Trends of aryl diazonium salt formation followed by Heck-Matsuda reaction of additional example using 3-(trifluoromethyl) aniline and 2-vinylbenzoic acid as starting materials, Figure S6: 3D surface for complete Heck-Matsuda reaction using 3-(trifluoromethyl) aniline and 2-vinylbenzoic acid as starting materials.
