# Supplementary material for: In Situ FTIR Spectroscopic Monitoring of the Formation of the Arene Diazonium Salts and Its Applications to the Heck–Matsuda Reaction
Source: Molecules. 2020 May 8;25(9):2199. doi: 10.3390/molecules25092199 (PMC7248935; doi:10.3390/molecules25092199)
Supplement: Supplementary file 1 [file molecules-25-02199-s001.pdf]

Supporting information

In situ FTIR Spectroscopic Monitoring of the Formation of the Arene Diazonium Salts and its Applications to Heck-Matsuda Reaction

K. Sateesh Reddy [1,2], Bandi Siva [3], S. Divya Reddy [4], N. Naresh Reddy [5], T.V. Pratap [1], B. Venkateswara Rao [2], Yi-An Hong [6], B. Vijaya Kumar [7], A. Krishnam Raju [7], P. Muralidhar Reddy [5,*] and Anren Hu [6,*]

[1]Technology Development Center, Custom Pharmaceutical Services, Dr. Reddy's Laboratories Ltd, Hyderabad 500049, India

[2]AU College of Engineering (A), Andhra University, Visakhapatnam 530003, India

[3]Laboratory for Bioanalytical Chemistry, Institute of Chemistry, University of Neuchatel, Avenue de Bellevaux 51, CH-2000 Neuchatel, Switzerland

[4]Department of Pharmacy, University College of Technology, Osmania University, Hyderabad, TS, India

[5]Department of Chemistry, University College of Science, Osmania University, Hyderabad-500007, TS, India

[6]Department of Laboratory Medicine and Biotechnology, College of Medicine, Tzu-Chi University, Hualien, Taiwan; amyhung840809@gmail.com (YH); anren@gms.tcu.edu.tw (AH)

[7]Department of Chemistry, Nizam College, Osmania University, Hyderabad 500001, TS, India

*Corresponding author(s): pmdreddy@osmania.ac.in, pmdreddy@gmail.com (PMR); Tel: +91-9848792423, anren@gms.tcu.edu.tw (AH) Tel.: +886-3-8565301 (ext. 2334 or 2335); Fax: +886-3-8571917 (AH.).

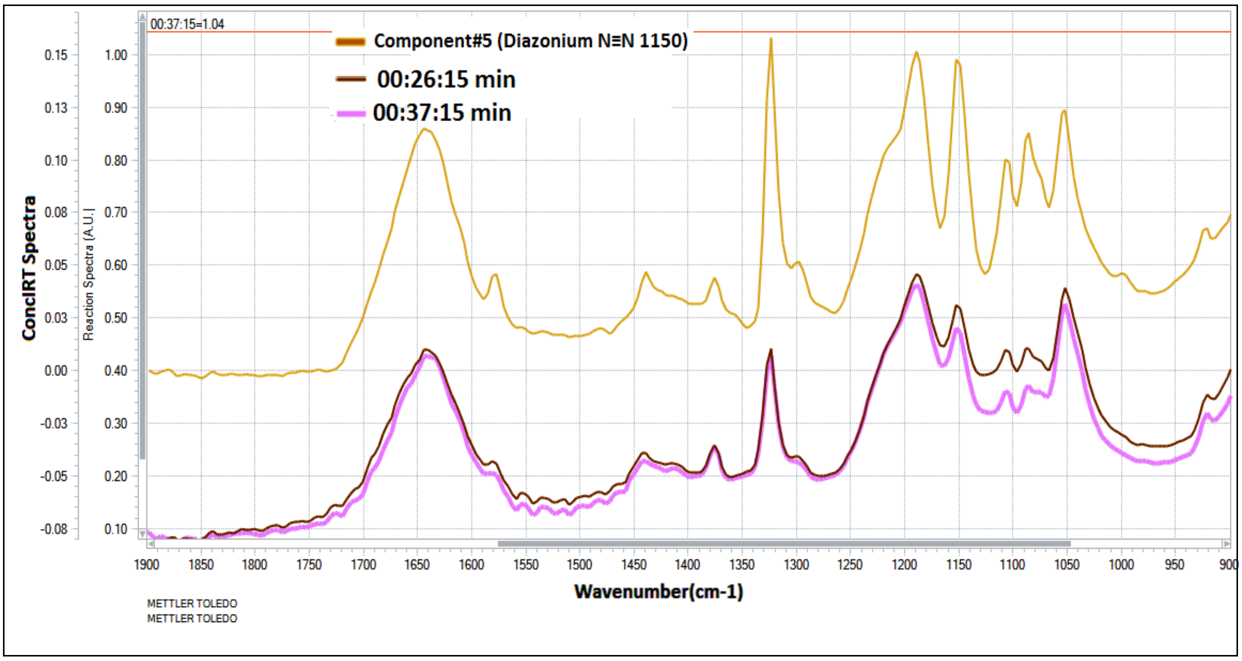

Figure S1: Comparison of IR spectra of Component #5 vs. 26:15 min spectra vs. 37:15 min spectra to understand the trend changes in figure 1. (Note: We suppose, these trend changes in red line between 20-30min and in blue line between 30-40 min in Figure 1 are due to sudden changes in concentration of diazonium component).

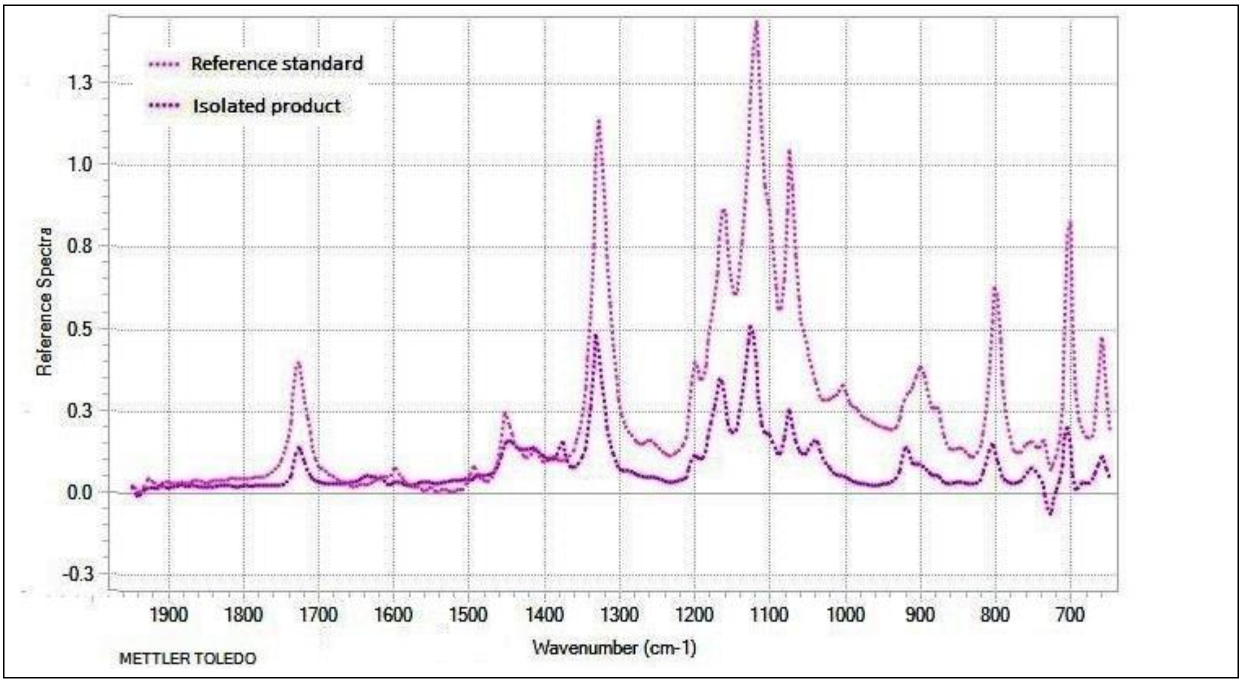

Figure S2: Comparison of IR spectra of standard vs isolated product

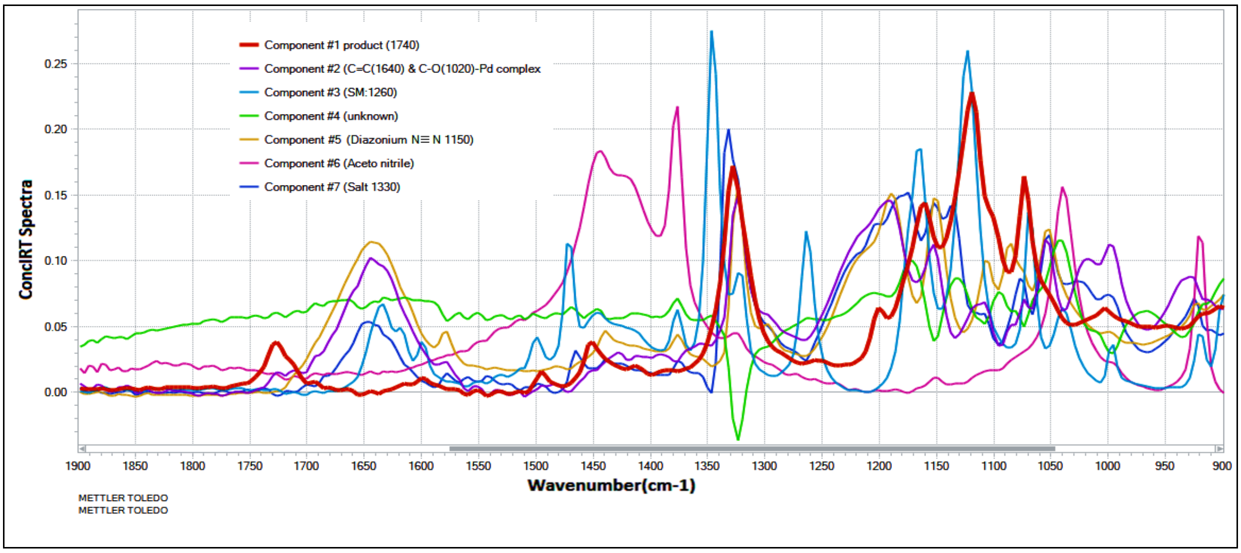

Figure S3: Complete IR spectra with all 7 components.

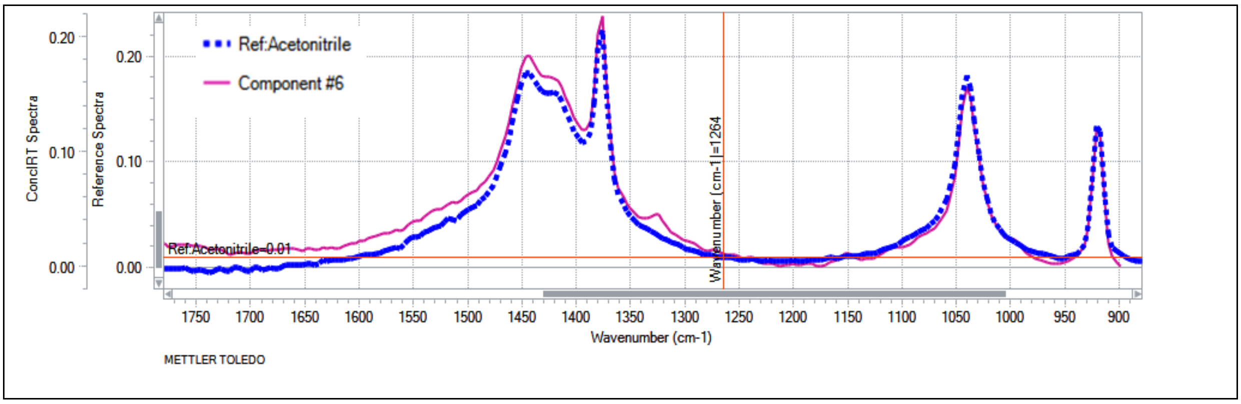

Figure S4: Comparison of IR spectra of Reference acetonitrile vs component 6

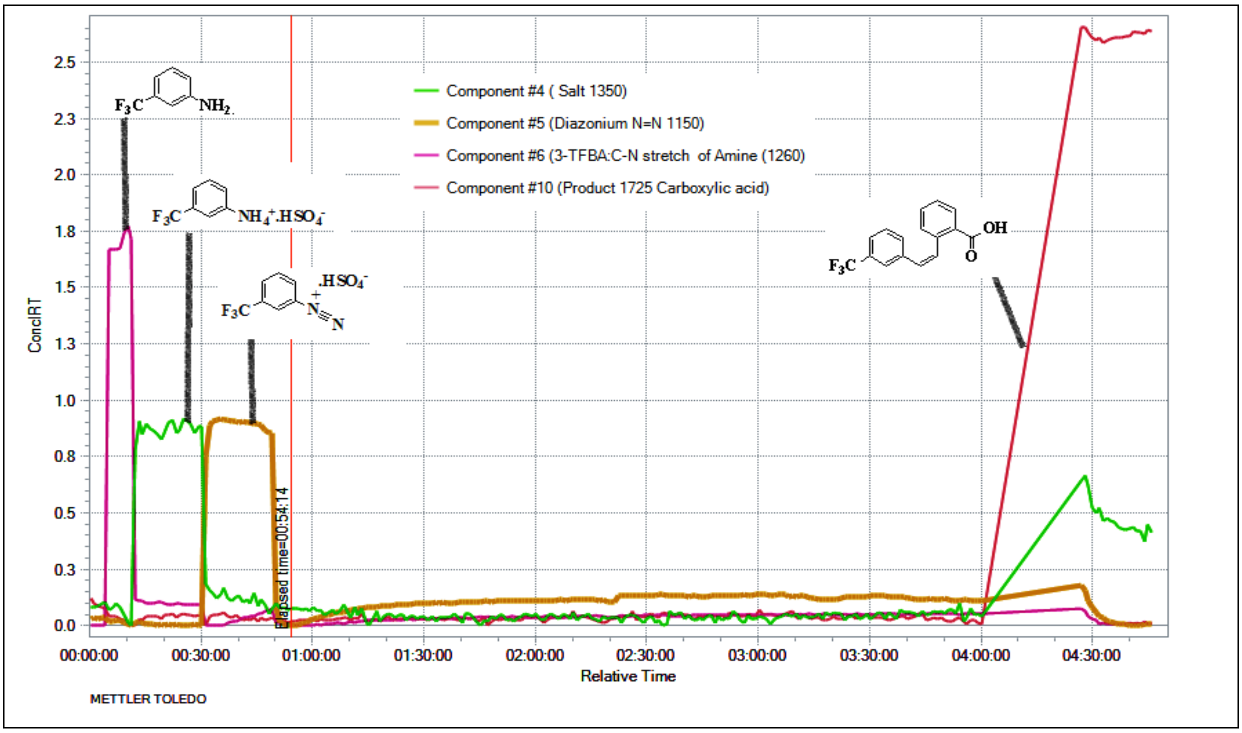

Figure S5. Trends of aryl diazonium salt formation followed by Heck-Matsuda reaction of additional example using 3-(trifloromethyl) aniline and 2-vinylbenzoic acid as starting materials. After addition of Palladium acetate and 2-vinylbenzoic acid to the diazonium salt(at 55min) reaction mass becomes heterogeneous and product concentration was reduced and water added at 4.0 hr and reaction mass becomes homogeneous and product shown with better ConcIRT value.

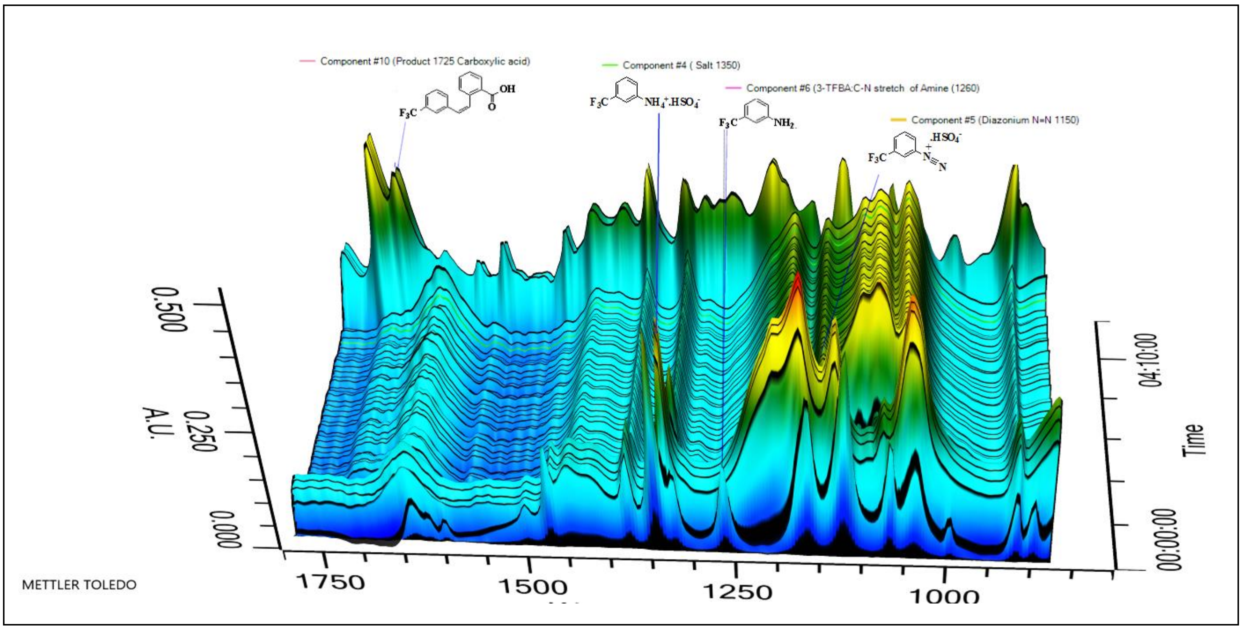

Figure S6. 3D surface for complete Heck-Matsuda reaction using 3-(trifloromethyl) aniline and 2-vinylbenzoic acid as starting materials
